# Highly Divergent T-cell Receptor Binding Modes Underlie Specific Recognition of a Bulged Viral Peptide bound to a Human Leukocyte Antigen Class I Molecule*[Fn FN2]

**DOI:** 10.1074/jbc.M112.447185

**Published:** 2013-04-08

**Authors:** Yu Chih Liu, John J. Miles, Michelle A. Neller, Emma Gostick, David A. Price, Anthony W. Purcell, James McCluskey, Scott R. Burrows, Jamie Rossjohn, Stephanie Gras

**Affiliations:** From the ‡ Department of Biochemistry and Molecular Biology, School of Biomedical Sciences, Monash University, Clayton 3800, Australia,; the § Queensland Institute of Medical Research and Australian Centre for Vaccine Development, Brisbane 4006, Australia,; the ¶ Institute of Infection and Immunity, Cardiff University School of Medicine, Heath Park, Cardiff CF14 4XN, Wales, United Kingdom,; the ‖ Human Immunology Section, Vaccine Research Center, NIADD, National Institutes of Health, Bethesda, Maryland 20892, and; the ** Department of Microbiology and Immunology, University of Melbourne, Parkville 3010, Australia

**Keywords:** Major Histocompatibility Complex (MHC), Structural Biology, T-cell Receptor, Viral Immunology, X-ray Crystallography

## Abstract

Human leukocyte antigen (HLA)-I molecules can present long peptides, yet the mechanisms by which T-cell receptors (TCRs) recognize featured pHLA-I landscapes are unclear. We compared the binding modes of three distinct human TCRs, CA5, SB27, and SB47, complexed with a “super-bulged” viral peptide (LPEPLPQGQLTAY) restricted by HLA-B*35:08. The CA5 and SB27 TCRs engaged HLA-B*35:08^LPEP^ similarly, straddling the central region of the peptide but making limited contacts with HLA-B*35:08. Remarkably, the CA5 TCR did not contact the α1-helix of HLA-B*35:08. Differences in the CDR3β loop between the CA5 and SB27 TCRs caused altered fine specificities. Surprisingly, the SB47 TCR engaged HLA-B*35:08^LPEP^ using a completely distinct binding mechanism, namely “bypassing” the bulged peptide and making extensive contacts with the extreme N-terminal end of HLA-B*35:08. This docking footprint included HLA-I residues not observed previously as TCR contact sites. The three TCRs exhibited differing patterns of alloreactivity toward closely related or distinct HLA-I allotypes. Thus, the human T-cell repertoire comprises a range of TCRs that can interact with “bulged” pHLA-I epitopes using unpredictable strategies, including the adoption of atypical footprints on the MHC-I.

## Teaser

**Background:** The mechanisms by which T cell receptors (TCRs) engage lengthy peptides bound to human leukocyte antigens (HLA) is unclear.

**Results:** We have determined the structures of two TCRs binding to a 13-residue bulged peptide presented by HLA-B*35:08.

**Conclusion:** TCRs can adopt markedly differing docking strategies upon engaging lengthy bulged peptides.

**Significance:** The human T-cell repertoire is sufficiently robust to deal with viral determinants of atypical length.

## Introduction

Clonally distributed αβ T-cell receptors (TCRs)^8^ on CD8^+^ cytotoxic T lymphocytes (CTLs) specifically recognize peptide (p) fragments, generally between 8 and 10 amino acids in length, presented by major histocompatibility complex class I (MHC-I) molecules expressed on the surface of all nucleated cells (1). TCR recognition of pMHC-I complexes is a key event in cellular immunity that is central to thymic selection (2), the lysis of pathogen-infected cells and the eradication of cancerous tissue. In general, TCR recognition is genetically restricted to self-MHC molecules, although the underlying basis of MHC restriction remains unclear (3–5). Puzzlingly, a relatively high frequency of T-cells break the “MHC restriction code” and recognize non-self (“allo”) human leukocyte antigen class I (HLA-I) molecules (6). The molecular mechanisms underpinning T-cell alloreactivity, a cause of T-cell-mediated transplant rejection, are beginning to emerge (7–9).

Structural studies have shed insight on the nature of the TCR-pMHC-I interaction (for recent reviews, see Refs. 3–5, 10, and 11). The TCR comprises six complementarity-determining regions (CDRs), whereby the germline-encoded CDR1/2 loops arise from the *TRAV* or *TRBV* genes (12), whereas the CDR3 loops lie at the V, (D), and J region junction, from which greater diversity is generated by random nucleotide (N) deletions and non-templated additions. TCRs can bind differing and identical pMHC-I epitopes using a range of docking modes (5, 7, 13). Within such TCR-pMHC-I complexes, the CDR loops of the TCR engage pMHC-I to varying extents (14). Despite these variations on a theme, a broad consensus TCR-pMHC-I footprint is conserved in which the Vα and Vβ domains of the TCR sit over the MHC-I α2- and α1-helices, respectively. The short antigenic peptide fragments generally protrude minimally from the MHC-I cleft, such that the TCR mostly contacts the MHC-I molecule. Nevertheless, associated mutagenesis studies in a number of TCR-pMHC-I systems have shown that generally only a few residues of the MHC-I (termed the “hot spots”) contribute significantly to the energetics of the TCR-pMHC-I interaction; moreover, such hot spots can vary between different TCR-pMHC-I systems (15–21). Such investigations highlight the peptide-centric nature of the TCR-pMHC-I interaction.

Although the N- and C-terminal ends of the MHC-I binding cleft constrain the majority of peptides to 8–10 amino acids in length, longer peptides are known to bind MHC-I (22–28). Indeed, such “atypically” long peptides can represent up to 10% of the peptide repertoire bound to MHC-I (29), and are known to play important roles in aberrant and protective immunity (30). Such peptides frequently bulge away from the antigen (Ag)-binding cleft, and can either exhibit substantial flexibility (23–25, 28) or adopt a more fixed conformation (25, 27, 31). The extent to which TCRs can accommodate such featured pMHC-I landscapes is unclear. Presently, TCRs have been shown to adopt two distinct strategies to ligate bulged pMHC-I epitopes. Namely, some TCRs can “flatten” the flexible and bulged peptide to enable an MHC-I-restricted response (32), whereas others form a more peptide-centric view and make very limited contacts with the MHC-I molecule (33). It is unclear whether other mechanisms might enable productive TCR recognition of atypical pMHC-I landscapes.

The recognition of a 13-amino acid (aa) determinant (LPEPLPQGQLTAY, termed “LPEP”) from Epstein-Barr virus, restricted by HLA-B*35:08, represents a well described example of how the T-cell repertoire, isolated from Epstein-Barr virus^+^ individuals (22), responds to a bulged pHLA-I epitope. Namely, the immune response to HLA-B*35:08^LPEP^ is underpinned by biased TCR usage in which the cognate repertoire is characterized by the dominant selection of TRBV5–6, 6–1, or 7–2 in distinct HLA-B*35:08 donors (33). In addition, the TCR α-chain is highly restricted, with TRAV19*01 and TRAJ34 found in nearly all HLA-B*35:08^LPEP^-specific TCRs. Previously, we determined the structure of one prototypical TCR, termed SB27 (TRAV19*01-TRAJ34*01-TRBV6–1*01) in complex with HLA-B*35:08^LPEP^ (33). Here, the SB27 TCR made a limited footprint on HLA-B*35:08, yet contacted the peptide extensively. The peptide centricity of the interaction was further emphasized by an alanine-scanning mutagenesis study at the SB27 TCR-HLA-B*35:08^LPEP^ interface (15). Although the SB27 TCR cross-reacted poorly with the closely related HLA allomorph, HLA-B*35:01, it nevertheless, alloreacted with HLA-B*44:02 (33), which differs from HLA-B*35:08 by 16 amino acids (supplemental Fig. 1). The TCR α- and β-chains of the SB27 TCR mediated many contacts with the bulged viral peptide. How different TCRs with different gene architecture bind the same HLA-B*35:08^LPEP^ epitope, and how these binding modes impact their alloreactivity profiles, is unknown.

Here, we assessed the impact of TCR α- and β-chain usage on the recognition of an atypical pHLA-I landscape. We show that alternate TCR architecture modifies the fine specificity toward the viral peptide and the pattern of alloreactivity, and can markedly alter the mode of cognate recognition. Notably, a unique docking mode was observed, in which the TCR does not contact the prominently exposed region of the viral peptide. Instead, contacts with HLA-I are maximized via an extreme N-terminal footprint, which enables the TCR to recognize a distinct feature of the MHC that has not been observed in previous TCR-pMHC-I structures.

## EXPERIMENTAL PROCEDURES

### 

#### 

##### Analysis of TCR Gene Expression

Unbiased amplification of all expressed *TRB* or *TRA* gene products from T-cell clones was conducted using template-switch anchored RT-PCRs incorporating a 3′ TRB constant region primer (5′-TGCTTCTGATGGCTCAAACACAGCGACCT-3′) or a 3′ TRA constant region primer (5′-AATAGGCAGACAGACTTGTCACTGGA-3′), respectively. Amplicons were subcloned, sampled, Sanger sequenced, and analyzed as described previously (34).

##### Cytotoxicity Assay

**FIGURE 1.**
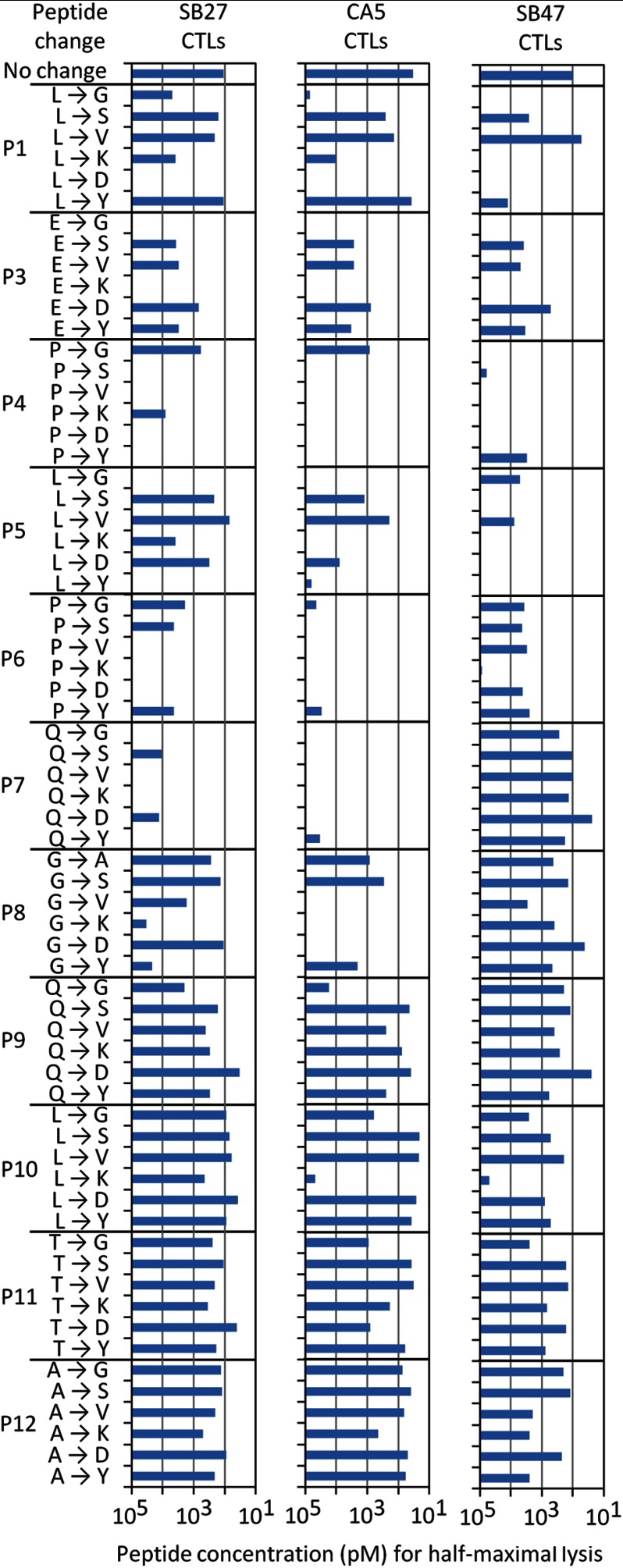
**Antigen specificity of the SB27, CA5, and SB47 TCRs.** Dose-response analysis of LPEP peptide analogues carrying single amino acid substitutions was conducted using chromium release assays to assess recognition by the SB27, CA5, and SB47 CTL clones. Peptide concentrations required to reach half-maximal lysis are shown. Some of the experiments with the SB27 clone were published previously and are shown here for comparison (33).

CTL clones were assayed in duplicate over a period of 5 h against peptide-pulsed ^51^Cr-labeled target cells. The target cells used to generate the data shown in Fig. 1 were HLA-B*35:08^+^ peripheral blood mononuclear cells expanded with phytohemagglutinin and propagated in IL-2-containing medium for up to 8 weeks. Percentage of specific lysis was calculated, and the peptide concentration required for half-maximum lysis was determined from dose-response curves. Peptides were synthesized by Mimotopes. A β scintillation counter (Topcount Microplate; Packard Instrument) was used to measure ^51^Cr levels in assay supernatant samples. The mean spontaneous lysis for targets in culture medium was always <20% and variation around the mean specific lysis was <10%. Normal lymphoblastoid cell lines and the C1R HLA-deficient mutant lymphoblastoid cell line were also used as targets, with or without transfection of the gene encoding HLA-B*44:02 (33).

##### Intracellular IFN-γ Staining

CTL clones were tested against a range of antigen-presenting cells and assayed for intracellular expression of IFN-γ by flow cytometry using the BD Cytofix/Cytoperm kit (BD Biosciences) according to standard protocols. Briefly, clones were incubated with antigen-presenting cells at a stimulator to responder ratio of 1:2 for 4 h in the presence of brefeldin A (BioLegend). For peptide titration experiments, T2 cells were incubated with various concentrations of LPEP peptide for 1 h and then washed thoroughly prior to incubation with CTL clones. After stimulation, cells were stained with Live/Dead Fixable Aqua Dead Cell Stain (Invitrogen) and anti-CD8-Cy5.5-PerCP mAb (BioLegend) for 30 min at 4 °C. Cells were then washed, fixed, permeabilized, and labeled with anti-IFN-γ-antigen-presenting cell mAb (BioLegend) for 30 min at 4 °C. After a further wash, samples were collected using a FACSCanto II flow cytometer (BD Biosciences). Data were analyzed with FlowJo software (Tree Star). Lymphocytes were identified based on light scatter, then live CD8^+^ T-cells were selected and IFN-γ^+^ gates were drawn based on unstimulated controls.

##### Protein Expression, Refolding, and Purification

The production of TCRs and pHLA-I molecules was performed as described previously (15). Briefly, individual chains of the CA5 and SB47 TCR genes were codon optimized for bacterial expression and cloned into the pET30 vector. Plasmids containing TCR chains were transformed into *Escherichia coli* BL21 cells for expression. Inclusion bodies were isolated and solubilized in urea buffer (8 m urea, 20 mm Tris-HCl, pH 8, and 1 mm DTT) before injecting into refolding buffer. The refolding procedure lasted 2 days before the samples were dialyzed against 10 mm Tris buffer (pH 8) three times daily at 4 °C. Dialyzed samples were loaded sequentially onto DEAE cellulose, size-exclusion, hydrophobic interaction, and anion exchange columns to obtain pure proteins. Protein size and purity were assessed by SDS-PAGE.

##### Thermal Stability Assay

Thermal stability assays of HLA-B*35:08 mutants in complex with the LPEP peptide were performed with the Real Time Detection instrument (Corbett RotorGene 300) as described previously (22). The results are summarized in supplemental Table S1.

##### Crystallization, Data Collection, and Structure Determination

TCR-pHLA-I ternary complexes were obtained by mixing the purified TCR and pHLA-I proteins at a 1:1 molar ratio. Initial crystallization trials were performed using the Monash Macromolecular Crystallization Facility Platform. Optimization trials were conducted via the hanging-drop vapor diffusion method whereby 1 μl of protein and 1 μl of the reservoir solutions were mixed accordingly. The CA5 TCR-HLA-B*35:08^LPEP^ complex crystals were obtained at 20 °C, using a protein concentration of 10 mg/ml in a reservoir solution containing 0.1 m sodium cacodylate (pH 6.7), 0.2 m potassium iodide, and 16% PEG 3350, with crystals of SB27 TCR-HLA-B*35:08^LPEP^ as cross-seeds. Crystals of SB47 TCR-HLA-B*35:08^LPEP^ were produced at 20 °C, using a protein concentration of 9 mg/ml in a reservoir solution containing 10 mm HEPES (pH 7.5), 0.2 m sodium tartrate, and 12% PEG 10K. Crystals of the TCR-pHLA-I complexes were soaked with the reservoir solution with increased percentages of PEG before being flash-frozen in liquid nitrogen. Frozen crystals were taken to the Australia Synchrotron, Melbourne, and datasets were collected with an ADSC-Quantum 315r CCD detector on the MX2 beamline at 100 K. Both datasets were processed and scaled with the XDS program (35). The structures were determined by molecular replacement (36) using the published SB27 TCR (PDB code 2AK4 (33)) and HLA-B*35:08 minus the peptide (PDB code 1ZHK (22)) as starting search models, whereby the HLA-I was located initially within the asymmetric unit, followed by the TCR. Manual building of the models was conducted with the COOT program (37) and structural refinements were conducted via Phenix (38) and Buster (39) with maximum-likelihood refinement. The TCRs were numbered according to the International Immunogenetics Information System unique numbering system (12), whereby the CDR1 loops start at residue 27, the CDR2 loops start at residue 56, and the CDR3 loops start at residue 105. The final models were validated using the Protein Data Bank validation Web site, and submitted to the Protein Data Bank database. All molecular graphic representations were created using PyMol (40).

##### Surface Plasmon Resonance

Surface plasmon resonance experiments were conducted using a BIAcore 3000 instrument at 25 °C with HBS-EP buffer (10 mm HEPES, pH 7.4, and 150 mm NaCl) containing 0.005% surfactant P20 and 1% bovine serum albumin to prevent nonspecific binding. The conformation-specific antibody 12H8 (17), which recognizes a conformational epitope within the TCR constant domain, was coupled to the surface of a research grade CM5 sensorchip using a standard amine-coupling protocol. Approximately 200–400 response units of each TCR (SB27, CA5 and SB47) was coated onto independent flow cells; the first flow cell was left empty as a negative control. Various concentrations (0.78–200 μm) of the analytes (either HLA-B*35:01^LPEP^ or HLA-B*35:08^LPEP^) were passed over all flow cells. Experimental data were analyzed using the BIAevaluation program (version 3.1) assuming the 1:1 Langmuir binding model with drifting baseline to determine the kinetic constants (Table 1). Using the same protocol, the HLA-B*35:08^LPEP^ mutants were flowed over the SB47 and SB27 TCRs at various concentrations (0.78–200 μm), and the equilibrium constant (*K*_deq_) was determined (supplemental Table S2). All experiments were performed at least twice (*n* = 2) in duplicate.

## RESULTS

### 

#### 

##### Fine Specificity of the CA5 and SB47 CTL Clones for the LPEP Peptide

The T-cell repertoire directed against the HLA-B*35:08-restricted ^52^LPEPLPQGQLTAY^64^ (“LPEP”) peptide derived from the Epstein-Barr virus lytic Ag BZLF1 exhibits biased TCR α-chain usage (TRAV19*01-TRAJ34*01), in which the α-chain is largely germline-encoded, possessing a small N-region that encodes a highly conserved ^94^Gly-Phe^95^ motif (33). In contrast, the TCR β-chain shows greater genetic variability (TRBV5–6, TRBV6–1, or TRBV 7–2) as well as differing CDR3β usage. For example, one HLA-B*35:08^+^ CTL clone, termed CA5, uses the same TCR α-chain and TRBV6–1*01 gene segment as the SB27 TCR, but a different Jβ gene segment (TRBJ1–1) (33). Consequently, their respective CDR3β loops differ markedly, whereby the CDR3β loop of the CA5 TCR (CASPGETEAF) is shorter than that of the SB27 TCR (CASPGLAGEYEQY). Interestingly, whereas the TRAV19*01-TRAJ34 α-chain dominated the CTL response to HLA-B*35:08^LPEP^, one CTL clone (termed SB47) exhibited a completely unique TCR architecture (TRAV39*01-TRAJ33-TRBV5–6*01-TRBJ2–7) (33). Accordingly, the SB27, CA5, and SB47 CTL clones enabled us to examine the impact of CDR3β variability and TCR gene usage on the response to HLA-B*35:08^LPEP^.

Previously, we demonstrated that the central region of the LPEP peptide (P4–P8) is critical for SB27 TCR recognition (33). To gain a detailed understanding of the fine specificity requirements of the CA5 and SB47 clones toward HLA-B*35:08^LPEP^, we performed cytotoxicity assays with single site substitutions along the solvent exposed residues of the LPEP peptide, using the SB27 clone as a control (Fig. 1). Each solvent-exposed position along the length of the peptide was mutated to one of 6 residues (Gly/Ala, Ser, Val, Lys, Asp, and Tyr). As expected from the crystal structure of the SB27 TCR-HLA-B*35:08^LPEP^ complex and previous fine specificity analyses (33), the SB27 clone showed negligible sensitivity toward substitutions at positions P9–P12, some sensitivity toward substitutions at P1, P3, P5, and P8, and substantial sensitivity toward substitutions at P4, P6, and P7. Overall, the fine specificity of the CA5 clone mirrored that of the SB27 clone, with substitutions at the N-terminal region of the epitope exhibiting a greater effect. Nevertheless, differences in the fine specificity patterns were observed. Namely, whereas the SB27 clone tolerated the substitution of P8-Gly to Val or Asp, these replacements were not tolerated by the CA5 clone (Fig. 1). In addition, the CA5 clone showed a heightened sensitivity at P4–P7 in comparison to the SB27 clone. Accordingly, differing CDR3β usage subtly affects the fine specificity profiles of the SB27 and CA5 clones.

Next, we examined the fine specificity of the SB47 clone toward the LPEP peptide. Substitutions at P7–P12 did not greatly impact on SB47 recognition, and a number of substitutions at P6 were also tolerated (Fig. 1). Thus, the SB47 clone was more tolerant to P6–P7 substitutions compared with the SB27 and CA5 clones. Additionally, the SB47 clone was considerably more sensitive at P1 and P5 compared with the SB27 and CA5 clones. These observations suggest that the fine specificity profile of the SB47 clone for the LPEP peptide is more N terminally focused than that of the SB27 and CA5 clones, in turn suggesting fundamentally different docking modes.

##### The Impact of HLA Polymorphism on T-cell Specificity

**FIGURE 2.**
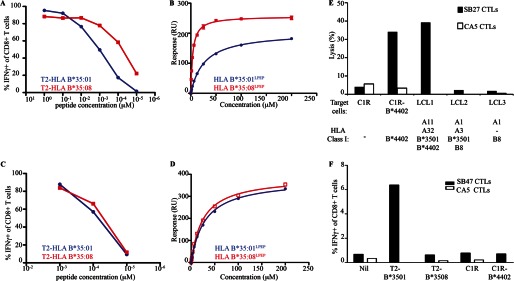
**Cross-reactivity of the SB27, CA5, and SB47 TCRs.** *A* and *C,* CD8^+^ T-cell activation was measured by intracellular cytokine staining for IFNγ after stimulation with peptide-pulsed T2 cells expressing either HLA-B*35:08 or HLA-B*35:01 as indicated. Experiments were performed twice with similar results. *B* and *D,* surface plasmon resonance experiments showing CA5 (*B*) and SB47 (*D*) TCR binding to HLA-B*35:08 and HLA-B*35:01 presenting the LPEP peptide. Data represent the concentration *versus* response unit curve derived from two experiments. *E* and *F,* HLA-restricted recognition and alloreactivity of the SB27, CA5, and SB47 CTL clones. *E,* specific lysis of target cells expressing different HLA molecules by SB27 and CA5 CTLs. The HLA^−^ cell line C1R was included as a control. *F,* activation of CA5 and SB47 CTLs after stimulation with T2 cells expressing HLA-B*35 allomorphs, parental C1R cells, or C1R cells expressing HLA-B*44:02.

The HLA-B*35:01/08 molecules differ only by a single residue, which is buried inside the Ag-binding cleft (Arg^156^ and Leu^156^ in HLA-B*35:08 and HLA-B*35:01, respectively (supplemental Fig. S1)). To dissect the fine preferences for MHC restriction exhibited by the CA5 and SB47 TCRs, we compared reactivity of the CA5 and SB47 CTL clones toward HLA-B*35:08 and HLA-B*35:01 targets presenting the LPEP peptide (Fig. 2). Although the CA5 CTL clone recognized both HLA allomorphs when high peptide concentrations were added, it preferentially recognized HLA-B*35:08^LPEP^ when the peptide was limited (Fig. 2*A*), in a similar manner to the SB27 clone. Next, we undertook surface plasmon resonance analysis of the recombinant CA5 and SB27 TCRs to establish their affinities for HLA-B*35:08^LPEP^ and HLA-B*35:01^LPEP^ (Fig. 2*B* and supplemental Fig. S2). The affinity values obtained for the SB27 TCR were consistent with those published previously (Table 1) (33), with a 4-fold decrease in the affinity for HLA-B*35:01^LPEP^ (*K*_deq_ = 52.25 ± 4.88 μm) compared with the HLA-B*35:08^LPEP^ complex (*K*_deq_ = 12.15 ± 0.35 μm). The affinity of the CA5 TCR toward HLA-B*35:08^LPEP^ (*K*_deq_ = 3.75 ± 0.01 μm) was ∼4-fold higher than that of the SB27 TCR (*K*_deq_ = 12.15 ± 0.35 μm), which was attributable to faster association and slower dissociation constants (CA5: *k*_on_ = 50.80 ± 8.06 10^4^ m^−1^ s^−1^, *k*_off_ = 0.19 ± 0.02 s^−1^; SB27: *k*_on_ = 10.05 ± 0.92 10^4^ m^−1^ s^−1^, *k*_off_ = 0.11 ± 0.01 s^−1^) (Table 1). In agreement with the cytotoxicity data, the affinity of the CA5 TCR toward HLA-B*35:01^LPEP^ (*K*_deq_ = 27.10 ± 0.42 μm) was notably weaker compared with HLA-B*35:08^LPEP^, which was due to slower association and faster dissociation rates upon HLA-B*35:01^LPEP^ binding (Fig. 2*B*, supplemental Fig. S2, and Table 1). Accordingly, the SB27 and CA5 TCRs exhibited similar patterns of cross-reactivity between HLA-B*35:01/08^LPEP^, although the CA5 TCR bound with higher affinity to these HLA allomorphs.

**TABLE 1:** **Surface plasmon resonance experiments for HLA-B35^LPEP^ and the CA5, SB27, and SB47 TCRs** The experiments were conducted at least twice (*n* = 2) in duplicate and the values represent the mean ± S.E.

Immobilized TCR	Analyte	*K*_deq_	*K*_on_	*K*_off_	*t*½*^a^*	*K_dcalc_*
		μ*m*	× *10^4^m*^−*1*^ *s*^−*1*^	*s*^−*1*^	*s*	μ*m*
CA5	HLA-B*35:08^LPEP^	3.75 ± 0.01	50.80 ± 8.06	0.19 ± 0.02	3.62 ± 0.32	3.78 ± 0.95
SB27	HLA-B*35:08^LPEP^	12.15 ± 0.35	10.05 ± 0.92	0.11 ± 0.01	6.21 ± 0.55	11.14 ± 0.03
SB47	HLA-B*35:08^LPEP^	25.00 ± 0.28	15.05 ± 5.16	0.30 ± 0.01	2.33 ± 0.05	19.77 ± 6.75
CA5	HLA-B*35:01^LPEP^	27.10 ± 0.42	34.45 ± 6.01	0.67 ± 0.16	1.07 ± 0.26	19.38 ± 1.34
SB27	HLA-B*35:01^LPEP^	52.25 ± 4.88	ND*^b^*	ND*^b^*	ND*^b^*	ND*^b^*
SB47	HLA-B*35:01^LPEP^	29.25 ± 0.07	15.50 ± 0.03	0.33 ± 0.04	2.15 ± 0.24	20.9 ± 1.99

*^a^ t*½ = 0.693/*K*_deq_.

*^b^* ND, not determined.

In contrast to the SB27 and CA5 CTLs, the SB47 clone recognized the LPEP peptide presented by HLA-B*35:08 and HLA-B*35:01 equally well (Fig. 2*C*). To assess whether the affinity of the SB47 TCR was affected by the HLA-B*35:01/08 polymorphism, we quantified SB47 TCR binding to HLA-B*35:08^LPEP^ and HLA-B*35:01^LPEP^. The affinity of the SB47 TCR for HLA-B*35:08^LPEP^ (*K*_deq_ = 25.00 ± 0.28 μm) was lower compared with the CA5 and SB27 TCRs, which was due to a faster dissociation rate (*k*_off_ = 0.30 ± 0.01 s^−1^) (Table 1). Furthermore, in contrast to the SB27 and CA5 TCRs, the SB47 TCR bound the HLA-B*35:08^LPEP^ and HLA-B*35:01^LPEP^ (*K*_deq_ = 29.25 ± 0.07 μm) complexes with similar affinities (Fig. 2*D*, supplemental Fig. S2, and Table 1). These findings are consistent with the functional data indicating that the SB47 TCR could not discriminate between the two closely related HLA-B35 allomorphs when bound to the LPEP peptide.

##### Unique Alloreactivity Footprints Across Clonotypes

The SB27 CTL clone alloreacts with HLA-B*44:02 presenting one or more unknown self-peptide(s) (Fig. 2*E*)(33). HLA-B*35:08 and HLA-B*44:02 differ by 16 residues, three of which (positions 80, 83, and 167) are solvent exposed (supplemental Fig. S1), yet are not involved in the SB27 TCR-HLA-B*35:08^LPEP^ interaction. Moreover, HLA-B*35:08 and HLA-B*44:02 are structurally very similar (root mean square deviation of the Ag-binding cleft is 0.4 Å), suggesting that the SB27 TCR could potentially bind both pHLA-I molecules with a similar docking mode. Next, we assessed whether the CA5 and SB47 clones could alloreact with HLA-B*44:02, using a CTL lysis assay against allogeneic cells in the absence of exogenous peptide. Surprisingly, the CA5 CTL clone did not alloreact with HLA-B*44:02 (Fig. 2*E*). Given that the CA5 and SB27 TCRs differ only in the CDR3β loop, which forms dominant interactions with peptide in the SB27 TCR-HLA-B*35:08^LPEP^ complex, this indicates that the observed differences in alloreactivity patterns are driven by peptide-centric interactions.

Similarly the SB47 clone did not alloreact with HLA-B*44:02 presenting self-peptides (Fig. 2*F*). However, the SB47 CTL clone did alloreact with HLA-B*35:01. This alloreactivity appeared to be dependent on one or more self-peptide(s) processed and presented independently of the transporter associated with antigen processing because the transporter associated with the antigen processing-deficient T2 cell line, transfected to express HLA-B*35:01, was recognized. In contrast, SB47 failed to recognize T2 cells transfected to express self-HLA-B*35:08. Given that the SB47 CTL clone did not distinguish between HLA-B*35:08 and B*35:01 presenting the LPEP viral peptide, this indicates that the nature of the self-peptide(s) dictates the HLA-B*35:01 alloreactivity. Accordingly, similar to the cognate interaction, the extent of alloreactivity across the three CTL clones is determined via a peptide-centric mechanism.

##### Structure of the CA5 TCR-HLA-B*35:08^LPEP^ Complex

**FIGURE 3.**
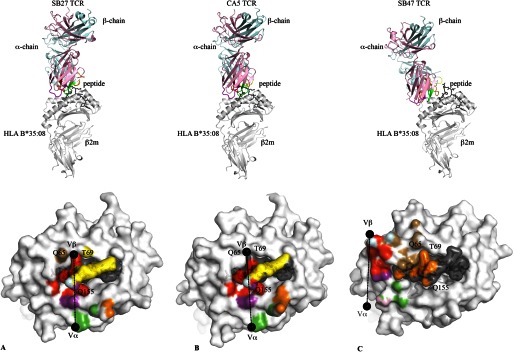
**Overview and structural footprints of the SB27, CA5, and SB47 TCRs.** Overview (*upper panels*) of the SB27 (*A*), CA5 (*B*), and SB47 (*C*) TCRs bound to HLA-B*35:08 (*white*) presenting the LPEP peptide (*black*). The color scheme is consistent across all panels and the orientation of HLA-B*35:08 is identical. TCR α- and β-chain framework residues are colored in *pale pink* and *cyan*, respectively. CDR1α, *purple*; CDR2α, *green*; CDR3α, *red*; CDR1β, *yellow*; CDR2β, *sand*; CDR3β, *orange*. The structural footprints (*lower panels*) of the SB27 (*A*), CA5 (*B*), and SB47 (*C*) TCRs onto HLA-B*35:08^LPEP^ are shown as surface representation with atoms colored based on the corresponding CDR loops that mediate the contacts. The *black spheres* represent the center of mass for the Vα and Vβ domains.

To understand how the CA5 TCR ligated to HLA-B*35:08^LPEP^, we determined its ternary complex and compared it to the SB27 TCR-HLA-B*35:08^LPEP^ complex (Fig. 3). The structure of the CA5 TCR-HLA-B*35:08^LPEP^ complex was solved at 2.3-Å resolution and refined to *R*_factor_/*R*_free_ values of 20.5 and 25.7%, respectively (Table 2). Unambiguous electron density was observed at the CA5 TCR-HLA-B*35:08^LPEP^ interface (supplemental Fig. S3, *A* and *B*). In contrast to the SB27 TCR-HLA-B*35:08^LPEP^ structure, in which two different orientations of the SB27 TCR were observed in the crystal lattice, the CA5 TCR-HLA-B*35:08^LPEP^ complex crystallized in a different space group, and only one ternary complex was present in the asymmetric unit.

**TABLE 2:** **Data collection and refinement statistics**

Structure	CA5 TCR-HLA-B*35:08^LPEP^	SB47 TCR-HLA-B*35:08^LPEP^
Resolution (Å)*^a^*	19.8-2.3 (2.5-2.3)	168-2 .8 (2.9-2.8)
Space group	*P*2_1_	*I*4
Temperature	100 K	100 K
Unit-cell parameters (Å)	55.10, 78.41, 105.34	237.60, 237.60, 61.20
(°)	β = 93.06	
No. observations	253,487 (56298)	299,703 (31899)
No. unique reflections	39,873 (8770)	42,851 (4208)
Completeness (%)	99.8 (100)	99.9 (100)
*R*_merge_*^b^* (%)	10.0 (42.5)	14.5 (47.6)
〈*I*/s(*I*)〉	13.2 (3.98)	9.59 (3.45)
Multiplicity	6.3 (6.4)	7.0 (7.5)

**Refinement statistics**		
Non-hydrogen atoms		
Protein	6727	6707
Water	132	124
*R*_factor_ *^c^*(%)	20.5	20.0
*R*_free_ (%)	25.7	23.3
Root mean square deviations from ideality		
Bond lengths (Å)	0.008	0.012
Bond angles (°)	1.08	1.21
Ramachandran plot (%)		
Most-favored region	91.2	91.4
Allowed region	8.2	8.4
Generously allowed region	0.4	0.2

*^a^* Values in parentheses represent the highest-resolution shell.

*^b^ R*_merge_ = Σ|*I_hkl_* − 〈*I_hkl_* 〉|/Σ*I_hkl_*.

*^c^ R*_factor_ = Σ*_hkl_* ‖*F_o_*| − |*F_c_*‖/Σ*_hkl_*|*F_o_*| for all data except 5%, which were used to calculate *R*_free_.

The CA5 TCR-HLA-B*35:08^LPEP^ complex overlaid closely with the SB27 TCR-HLA-B*35:08^LPEP^ complex (root mean square deviation of 1.65 Å over the entire complex) (Fig. 3, *A* and *B*). As such, the CA5 TCR docked orthogonally (93°) to the long axis of the binding cleft of HLA-B*35:08 (Fig. 3*B*). The total buried surface area (BSA) at the CA5 TCR-HLA-B*35:08^LPEP^ interface (≈ 1800 Å^2^) was similar to that of the SB27 TCR-HLA-B*35:08^LPEP^ interface (≈ 1900 Å^2^), and the total number of contacts at the respective interfaces was similar (CA5 TCR: 142 van der Waals, 13 hydrogen bonds; SB27 TCR: 137 van der Waals, 16 hydrogen bonds, and 1 salt bridge). Moreover, the contributions from each of the CDR loops at the respective interfaces were similar, with the CDR1α, -2α, and -3α loops of the CA5 TCR contributing 13.0, 18.4, and 32.9% of the total BSA, respectively, and the CDR1β, -2β, and -3β loops of the CA5 TCR contributing 17.5, 1.7, and 15.6% of the total BSA, respectively. Accordingly, consistent with the biased TRAV19*01-TRAJ34*01 usage, the α-chain of the CA5 TCR dominated the interface (BSA 64.3%).

**FIGURE 4.**
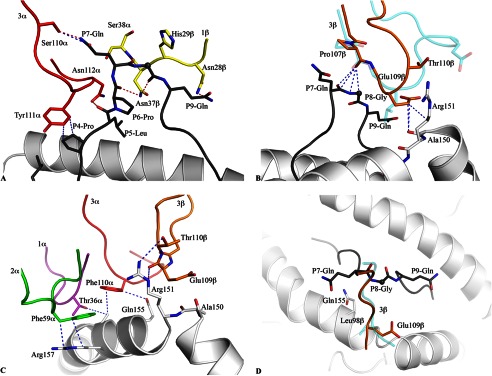
Contacts between the CA5 TCR and the HLA-B*35:08^LPEP^ complex. *A*, CA5 TCR-peptide interactions were mediated primarily via the CDR3α (*red*) and CDR1β (*yellow*) loops. *B,* structural superposition of the CDR3β loops of the CA5 TCR (*orange*) and SB27 TCR (*transparent cyan*) in the corresponding ternary complexes. The conserved TCR-peptide interaction, with the peptide in *black stick*, is highlighted and the specific TCR-HLA interaction mediated by the CA5 TCR is compared with the SB27 TCR. *C,* conserved TCR-HLA interactions observed in the CA5 and SB27 ternary complex structures. *D*, *top view* of the CDR3β loop interaction with the HLA-B*35:08^LPEP^ complex for the CA5 TCR (*orange*) superposed with the SB27 TCR (*transparent cyan*). This view shows the difference in orientation between the Glu^β109^ and Leu^β98^ side chains in the CA5 and SB27 TCRs, respectively. All structural representations follow the color scheme depicted in Fig. 3. *Blue dashed lines* represent van der Waals interactions; *red dashed lines* represent hydrogen-bond contacts; *spheres* represent the Cα atom of glycine residues.

Akin to the SB27 TCR, the CA5 TCR adopted the same “peptide-centric” docking onto the HLA-B*35:08^LPEP^ complex, with ∼50% of the BSA at the interface arising from the bulged peptide (Fig. 3*B*), which markedly contrasts with typical TCR-pMHC interactions involving peptides of canonical length (BSA 20%) (41). Furthermore, similar to the SB27 TCR interaction, the CA5 TCR did not deform the bulged peptide upon ligation, and the conformation of the peptide in the SB27 TCR and CA5 TCR ternary complexes was very similar. Within the CA5 and SB27 TCR complexes, the TCR-peptide interaction was mediated primarily via the CDR3α and CDR1β loops (15) (Fig. 4*A*). Specifically, the CDR1β loop of the CA5 and SB27 TCRs ran parallel to the bulged Ag, forming extensive interactions with residues from P6 to P9 of the peptide, whereas the CDR3α loops flanked primarily the ascending region of the LPEP peptide between residues P4 to P7 (Fig. 4*A*).

Despite the overall similarities between the CA5 TCR-HLA-B*35:08^LPEP^ and SB27 TCR-HLA-B*35:08^LPEP^ complexes, differences within the interfaces were apparent. First, the shape complementarity (42) at the CA5 TCR-HLA-B*35:08^LPEP^ interface (shape complementarity = 0.77) was moderately higher than that of the corresponding SB27 TCR interface (shape complementarity = 0.71), which correlated with the higher affinity of the CA5 TCR toward HLA-B*35:08^LPEP^. Second, in comparison to the SB27 TCR footprint, the CA5 TCR docked slightly differently onto HLA-B*35:08^LPEP^, with the most notable consequence being that the CA5 TCR did not directly contact any residues from the α1-helix of HLA-B*35:08 (Fig. 3*B*). Although the SB27 TCR contacts two positions on the α1-helix (65 and 69) (Fig. 3*A*), previous mutagenesis studies on the SB27 TCR-HLA-B*35:08^LPEP^ complex underscore the observation that the α1-helix of HLA-B*35:08 does not appreciably contribute to the TCR-HLA-B*35:08^LPEP^ interface of either the SB27 or CA5 TCRs (Fig. 3*B*). Conversely, residues within the α2-helix of HLA-B*35:08 define an energetic hot spot that underpins SB27 TCR recognition (15). Given that these residues, namely Arg^151^, Gln^155^, Arg^157^, and Ala^158^, are also contacted by the CA5 TCR, this suggests that a common hot spot within the α2-helix of HLA-B*35:08 drives the association with the SB27 and CA5 TCRs (supplemental Table S3, Fig. 4*C*).

##### CDR3β Loop-mediated Interactions

The SB27 and CA5 TCRs differ in their CDR3β loops (CA5 TCR, CASPGETEAF; SB27 TCR, CASPGLAGEYEQY), and the interactions mediated by this loop varied accordingly between the two ternary complexes. Within the SB27 TCR-HLA-B*35:08^LPEP^ complex, the CDR3β loop contacted the peptide and the α2-helix of HLA-B*35:08. Pro^β95^ solely interacted with the peptide, contacting the P7-Gln and P8-Gly residues located at the top of the bulged peptide (Fig. 4*B*). The SB27 CDR3β-HLA-B*35:08 interactions principally arose from Leu^β98^ forming a large number of van der Waals contacts with HLA-B*35:08 and Glu^β101^ salt bridging to Arg^151^. Although the conformation of the N-terminal region (^104^CASPG^108^) in the CA5 TCR CDR3β loop overlaid closely with the corresponding region of the SB27 CDR3β loop, the remainder of the CDR3β loop adopted a markedly different conformation and, as a consequence, the CDR3β-HLA-B*35:08 interactions differed (Fig. 4*B*). Namely, the salt bridge with Arg^151^ was absent in the CA5 TCR ternary complex; instead, Arg^151^ formed van der Waals contacts with Thr^β110^ (Fig. 4*C*). Additionally, Leu^β98^ in the SB27 TCR was replaced by Glu^β109^ in the CA5 TCR, whose side chain orientated away from the interface (Fig. 4*D*), with its aliphatic moiety packing against Ala^150^ and Arg^151^ (Fig. 4, *B* and *C*). As a consequence, although the CA5 Glu^β109^ did not directly contact the bulged peptide, its close proximity with P8-Gly (Fig. 4*D*) may explain why the CA5 CTL clone was more sensitive to P8 substitutions than the SB27 CTL clone (Fig. 1).

##### Overview of the SB47 TCR-HLA-B*35:08^LPEP^ Complex

To understand the shifted fine specificity profile of the SB47 TCR (Fig. 1), we determined the crystal structure of the SB47 TCR-HLA-B*35:08^LPEP^ complex. The structure was solved to 2.8-Å resolution and refined to *R*_factor_/*R*_free_ values of 20.0 and 23.3%, respectively (Table 2). Unambiguous electron density was observed at the SB47 TCR-HLA-B*35:08^LPEP^ interface (supplemental Fig. S3, *C* and *D*).

The SB47 TCR bound orthogonally onto HLA-B*35:08^LPEP^ with a docking angle of ∼87° (Fig. 3*C*), similar to the orientation of the CA5 and SB27 TCRs ligated to HLA-B*35:08^LPEP^ (Fig. 3). However, in stark contrast to the CA5 and SB27 TCRs docking modes, the SB47 TCR was positioned toward the very N-terminal end of the HLA-B*35:08 Ag-binding cleft, thereby essentially not contacting the prominent apex of the super-bulged peptide.

The total BSA at the SB47 TCR-HLA-B*35:08^LPEP^ interface was ≈2000 Å, moderately higher than the BSA of the SB27 and CA5 TCR-HLA-B*35:08^LPEP^ complexes, yet nevertheless, within the range of TCR-pMHC-I interactions determined to date (5). The greater BSA was consistent with an increased number of interactions observed at the SB47 TCR-HLA-B*35:08^LPEP^ interface (168 van der Waals, 16 H-bonds, and 3 salt bridges) compared with the respective CA5 and SB27 TCR-HLA-B*35:08^LPEP^ interfaces. However, unlike the centrally docked SB27 and CA5 TCRs, which made limited contacts with HLA-B*35:08, the extreme N-terminal positioning of the SB47 TCR allowed extensive interactions with the HLA-I molecule itself. Indeed, at this interface, the SB47 TCR interacted with the α1- and α2-helices (spanning residues 55–68 and 162–170, respectively). Consequently, the SB47 TCR-HLA-B*35:08 interactions (BSA 83%) were more prominent than the SB47 TCR-peptide contacts (BSA 17%) (Fig. 3*C*). Nevertheless, despite this more extensive HLA footprint, the affinity of the SB47 TCR for HLA-B*35:08^LPEP^ was weaker than that of the CA5 and SB27 TCRs (Table 1).

##### A New Footprint on HLA-I

As exemplified by the SB27 and CA5 TCR-HLA-B*35:08^LPEP^ complexes, a bulged and rigid peptide bound within the Ag-binding cleft acts a “hurdle” for the TCR to dock extensively onto the HLA itself. However, the SB47 TCR overcomes this hurdle by adopting a markedly shifted N-terminal footprint, whose center of gravity differs markedly (by 18 Å) compared with that of the SB27 TCR. Indeed, due to its extreme N-terminal footprint, the SB47 TCR did not contact position 155 of HLA-B*35:08 (Fig. 3*C*), a position that represents a TCR contact point in all TCR-pMHC-I structures determined to date (5, 14).

**FIGURE 5.**
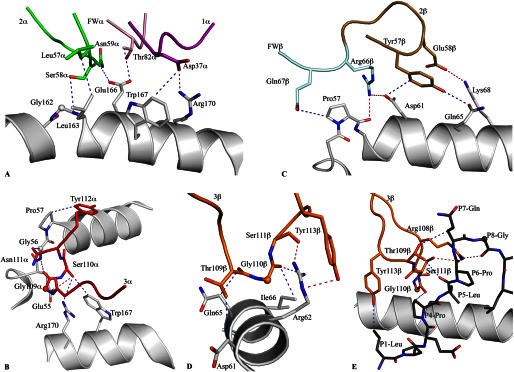
Contacts between the SB47 TCR and the HLA-B*35:08^LPEP^ complex. *A,* the germline-encoded SB47 Vα chain, including CDR1α, CDR2α, and framework residues, interacted extensively with the α2-helix of HLA-B*35:08. *B,* the CDR3α loop bridged between the α1- and α2-helices, contacting a loop region from Glu^55^ to Pro^57^ of HLA-B*35:08. *C,* the CDR2β loop and its neighboring framework residues sat directly above the N-terminal part of the α1-helix of HLA-B*35:08, making contacts between Pro^57^ and Lys^68^. *D,* the CDR3β loop contacted a small stretch of the α1-helix of HLA-B*35:08, including Gln^65^. *E*, the SB47 TCR interacted with the N-terminal region of the peptide, including P1-Leu and P4-Pro to P8-Gly, exclusively via the CDR3β loop. All structural representations follow the color scheme depicted in Fig. 3. *Blue dashed lines* represent van der Waals interactions; *red dashed lines* represent hydrogen-bond contacts; *spheres* represent the Cα atom of glycine residues.

Five of six SB47 CDR loops contacted HLA-B*35:08 (Fig. 3*C*, supplemental Table S4), with the CDR3α and CDR3β loops contributing most extensively to the interface (19.5 and 39.7% of the total BSA, respectively). Remarkably, only one HLA-B*35:08 contact point was shared between the SB47 and CA5 TCRs (Gly^162^), and the SB47 and SB27 TCRs (Gln^65^); moreover, different CDR loops were involved in these contacts (supplemental Table S4). The SB47 α-chain (BSA 44%) contacted both helices of HLA-B*35:08 (Fig. 3*C*). The role of the CDR1α loop (BSA 8.7%) was limited to Asp^α37^ salt bridging to Arg^170^, and forming van der Waals contacts with Trp^167^ (Fig. 5*A*). A stretch of residues, ^57^LSN^59^, from the CDR2α loop (BSA 12.2%), contacted Gly^162^, Leu^163^, and Glu^166^, the latter of which interacted with the framework residue, Thr^α82^ (Fig. 5*A*). The CDR3α loop formed an extended network of interactions with HLA-B*35:08, with its loop positioned orthogonally to the main axis of the α1-helix, contacting residues spanning positions 55 to 61, as well as interacting with Trp^167^ and Arg^170^ of the α2-helix (Fig. 5*B*). Of note, residues 55–57 of HLA-B*35:08 form a turn before the start of the α1-helix, and this region of the HLA-I has not been contacted by any TCR determined to date (Fig. 5*B*) (5).

The SB47 β-chain (BSA 56%) exclusively contacted the α1-helix via its CDR2β (BSA 8.6%) and CDR3β loops (supplemental Table S4). The interactions mediated via the CDR2β loop involved Tyr^β57^ and Glu^β58^, as well as two framework (FW) residues upstream of CDR2β, namely Arg^β66^ and Gln^β67^ (Fig. 5*C*). The CDR2β/FWβ interaction spanned from Pro^57^ to Lys^68^ and involved a number of van der Waals interactions and two salt bridges (Lys^68^ with Glu^β58^ and Asp^61^ with Arg^β66^) (Fig. 5*C*). The CDR3β loop was wedged between the ascending part of the bulged peptide (P4–P7) and Arg^62^ of the α1-helix. The non-germline-encoded residues Thr^β109^ and Gly^β110^ made van der Waals contacts with Gln^65^/Asp^61^ and Arg^62^/Ile^66^, respectively (Fig. 5*D*). The majority of the interactions between the CDR3β loop and the α1-helix were focused around Arg^62^, whose side chain was flanked by the main chain of the CDR3β loop and Tyr^β113^ (Fig. 5*D*). Collectively, the SB47 TCR adopted a markedly contrasting footprint onto HLA-B*35:08 compared with the SB27 and CA5 TCRs. This extreme N-terminal footprint is reminiscent of the way in which some autoreactive TCRs dock onto MHC-II (supplemental Fig. S2) (43–45), although N-terminal docking footprints have also been observed for antimicrobial TCR-pMHC-I complexes (13).

**FIGURE 6.**
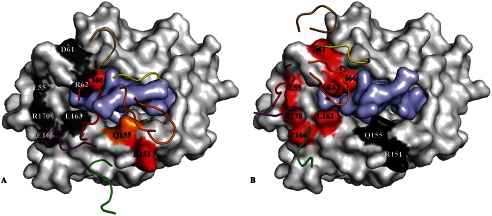
Energetic footprints of the SB27 and SB47 TCRs on the HLA-B*35:08^LPEP^ complex. *A* and *B,* surface representation of HLA-B*35:08 (*white*) with the LPEP peptide (*pale purple*). The energetic footprint is colored accordingly to the importance of each residue, with critical residues in *red* (*K*_deq_ decrease > 5-fold); important residue in *orange* (5-fold> *K*_deq_ decrease > 3-fold); *dark gray* indicates residues that were not important for the interaction. *Panel A* shows the SB27 TCR energetic footprint, and *panel B* shows the SB47 TCR energetic footprint. The CDR loops are represented in schematic format and colored according to the scheme used in Fig. 3.

To investigate further the importance of the N-terminal region of HLA-B*35:08 for SB47 TCR recognition, we performed alanine scanning mutagenesis in conjunction with surface plasmon resonance analyses. Of the N-terminal HLA-B*35:08 residues involved in the interaction with SB47 TCR, we mutated the following to alanine: Glu^55^, Asp^61^, Arg^62^, Ile^66^, Leu^163^, Glu^166^, and Arg^170^. We also included two control residues, namely Arg^151^ and Gln^155^, as they are not contacted by the SB47 TCR, yet they are important for SB27 TCR recognition (15). First, we tested the stability of each mutant via a thermal shift assay; only the I66A mutant decreased the stability of the pHLA-I complex significantly (by more than 10 °C) (supplemental Table S1) (22). Next, we performed surface plasmon resonance analyses against the panel of HLA-B*35:08^LPEP^ mutants (supplemental Table S2). The I66A mutant impacted on SB47 and SB27 TCR recognition, despite the absence of direct contacts with the SB27 TCR. This effect is most likely indirect, with Ile^66^ being important for maintaining pMHC structural integrity. The control mutations, including Arg^151^ and Gln^155^, both exhibited decreased affinities with the SB27 TCR yet had minimal impact on SB47 TCR recognition (supplemental Table 2A). The six other HLA-B*35:08 mutants all decreased SB47 TCR binding affinity by more than 7-fold, with minimal effects on SB27 TCR affinity (supplemental Table S2, Fig. 6). The energetic footprint of the SB47 TCR is in marked contrast to the corresponding energetic footprint for the SB27 TCR (15) (Fig. 6). Collectively, the mutagenesis data not only highlight the N terminally focused nature of the SB47 TCR but also, for the first time, illustrate the importance of the Glu^55^ and Asp^61^ HLA residues in enabling TCR recognition.

##### New TCR Footprint on the Bulged Peptide

Due to the distinct SB47 TCR footprint, its interaction with the bulged peptide differed from that of the SB27 and CA5 TCRs. Namely, the SB47 TCR formed limited contacts with the LPEP peptide, mediated exclusively via the CDR3β loop (Fig. 5*E*). The CDR3β loop sequence, ^108^RTGSTYE^114^, contacted the N-terminal part of the peptide, P1-Leu and P4-Pro, as well as the ascending part of the super-bulge, from P5-Leu to P8-Gly (Fig. 5*E*). The P1-Leu residue interacted with Tyr^β113^, and P4-Pro contacted the main chain of Gly^β110^-Ser^β111^, thereby providing a basis for the decreased CTL activity observed when these two positions were substituted in the LPEP peptide (Fig. 1). The interactions spanning P5-Leu to P8-Gly were mostly featured by main chain interactions arising either from the peptide or the CDR3β loop, with three H-bonds (Arg^108βNH^_2_ to P6-Pro^O^, P7-Gln^O^, P8-Gly; Gly^110βO^ to P5-Leu^N^; Ser^111βOγ^ to P5-Leu^O^, P6-Pro^O^) helping to drive the specificity of this interaction. Additionally, the P5-Leu side chain packed against Arg^β108^ and the main chain of the CDR3β loop. The P7-Gln and P8-Gly residues were mostly solvent exposed (Fig. 5*E*), thereby providing a basis for understanding why the SB47 TCR was tolerant to substitutions at these positions. Thus, by adopting an extreme N-terminal docking mode on HLA-B*35:08, the SB47 TCR does not contact the most prominent feature of the bulged peptide.

## DISCUSSION

The circulating TCR repertoire encompasses enormous diversity and enables adaptive immune responses to a universe of different pMHC complex antigens. For peptides of canonical length (8–10 residues) presented by MHC-I, a portrait of how closely related and distinct TCRs can interact with the same pMHC epitope has been established (5). In particular, for Vβ8.2^+^ TCRs, which are arguably the most intensively investigated (4), approximate common TCR-pMHC docking modes have prevailed, thereby suggesting a basis for MHC bias that largely arises from conserved germline-encoded interactions between the CDR2β loop and a given region of the MHC (46, 47). A wider range of docking footprints atop the same pMHC landscape is apparent when distinct TCR α- and/or β-chain usage is considered (13, 16, 48). Previously, we provided insight into how the T-cell repertoire can accommodate atypical peptides (>10 amino acids in length) bound to the MHC-I (32, 33). Two divergent TCR recognition modes were apparent. In one mode, the bulged peptide was flattened to enable a large and “standard” MHC footprint (32). In the other mode, a predominantly peptide-centric interaction with a rigid bulged peptide resulted in a very limited MHC-I footprint (33). Here, we provide structural and mechanistic data to explain how both closely related and distinct TCRs can interact with a “super-bulged” peptide presented by HLA-I.

The CA5 TCR is encoded by the same *TRAV-TRAJ-TRBV* gene combination as the archetypal SB27 TCR, with the differences being confined to their respective CDR3β loops. Accordingly, the overall TCR-HLA-B*35:08^LPEP^ footprints were similar. The SB27 TCR made limited contacts with the α1- and α2-helices of HLA-B*35:08. Nonetheless, the footprint was even more restricted with the CA5 TCR, which does not contact the α1-helix. Accordingly, despite a consensus docking mode between these two TCRs, the non-germline-encoded CDR3β loop affected MHC-driven contacts, whereas essentially preserving the extent of the peptide-TCR interactions. This highlights the peptide-centricity of this particular TCR-pMHC-I interaction and makes generalizations, such as two-step binding modes and exclusive germline-encoded MHC bias, difficult to reconcile in this system (49). Intriguingly, whereas the SB27 and CA5 TCRs both preferentially recognized HLA-B*35:08^LPEP^ over HLA-B*35:01^LPEP^, they nevertheless, showed different patterns of alloreactivity. Indeed, only the SB27 TCR alloreacted with HLA-B*44:02. Given the close structural homology between HLA-B*35:08 and HLA-B*4402, and the peptide-centric focus of these TCRs, these data suggest that peptide-centric molecular mimicry defines HLA-B*44:02 alloreactivity for the SB27 TCR and that the nature of the HLA-B*44:02-restricted self-peptide is non-permissive for CA5 TCR binding. In line with this view, fine specificity differences between SB27 TCR and the CA5 TCR with regard to the cognate LPEP peptide were also apparent.

Additionally, we determined the structure of a TCR that possessed markedly different *TRAV-TRAJ-TRBV* gene usage in comparison to the SB27 and CA5 TCRs, yet nevertheless, recognized the same HLA-B*35:08^LPEP^ complex. Remarkably, the SB47 TCR employed a peculiar and unprecedented binding mechanism to accommodate the bulged LPEP epitope. Namely, by establishing an extensive footprint on the extreme N-terminal end of HLA-B*35:08, the SB47 TCR essentially circumvented the most prominent feature of the bulged epitope. The CDR3β loop and, to a lesser extent, the CDR3α loop, dominated contacts with HLA-B*35:08^LPEP^, with the docking mode enabling a more HLA-centric view in comparison to the SB27 and CA5 TCRs. Moreover, the markedly shifted N-terminal footprint enabled the TCR to contact a region of MHC-I that had not been observed previously to mediate contacts with a TCR. Notably, this docking strategy also obviated TCR contacts with Gln^155^ from HLA-B*35:08, which is surprising given that position 155 has been contacted in every TCR-pMHC-I complex reported to date (5). The N-terminal docking mode explains why the SB47 TCR was able to bind both HLA-B*35:08^LPEP^ and HLA-B*35:01^LPEP^, as the point of difference between these two allomorphs resides within the α2-helical hinge, a region that is not contacted in the corresponding ternary complex. Although the SB47 TCR did not alloreact with HLA-B*44:02 and was not activated by HLA-B*35:08 presenting self-peptides, it nevertheless, alloreacted with HLA-B*35:01. Intriguingly, this implicates differences in the repertoire or conformation of self-peptides bound to HLA-B*35:01 *versus* HLA-B*35:08 in the HLA-B*35:01 alloreactivity of this TCR.

In summary, our data demonstrate that alternative TCR structures with unanticipated docking modes can contribute to T-cell-mediated immune recognition of a lengthy and rigid viral peptide bound to MHC-I. These unusual strategies not only illustrate the versatility of the T-cell repertoire, but also shape our understanding of MHC restriction and TCR alloreactivity.

## Supplementary Material

Supplemental Data

## Footnotes

8 The abbreviations used are:

TCR: T-cell receptor
BSA: buried surface area
CDR: complementarity-determining region
pMHC: peptide-major histocompatibility complex
CTL: cytotoxic T lymphocyte
HLA: human leukocyte antigen.
