# Supplementary material for: Highly Divergent T-cell Receptor Binding Modes Underlie Specific Recognition of a Bulged Viral Peptide bound to a Human Leukocyte Antigen Class I Molecule
Source: J Biol Chem. 2013 Apr 8;288(22):15442–54. doi: 10.1074/jbc.M112.447185 (PMC3668706; doi:10.1074/jbc.M112.447185)
Supplement: Supplemental Data [file supp_288_22_15442__index.html]

Highly divergent T-cell receptor binding modes underlie specific recognition of a bulged viral peptide bound to a HLA class I molecule — Highly Divergent T-cell Receptor Binding Modes Underlie Specific Recognition of a Bulged Viral Peptide bound to a Human Leukocyte Antigen Class I Molecule — T-cell Receptor Recognition of an Atypical pMHC — Supplemental Data 

# Highly Divergent T-cell Receptor Binding Modes Underlie Specific Recognition of a Bulged Viral Peptide bound to a Human Leukocyte Antigen Class I Molecule

## Supplemental Data

**Files in this Data Supplement:**

- Supplemental Data (.pdf, 3.0 MB)
