# Supplementary material for: Highly Divergent T-cell Receptor Binding Modes Underlie Specific Recognition of a Bulged Viral Peptide bound to a Human Leukocyte Antigen Class I Molecule
Source: J Biol Chem. 2013 Apr 8;288(22):15442–54. doi: 10.1074/jbc.M112.447185 (PMC3668706; doi:10.1074/jbc.M112.447185)
Supplement: Supplemental Data [file supp_M112.447185_jbc.M112.447185-1.pdf]

Supplementary Data

Figure S1. Polymorphic residues. (A) The HLA-B*35:08 molecule is represented in green cartoon, with HLA-B*35:01 in orange cartoon. The unique polymorphic residue at position 156 is represented in stick. (B) The graphic represents the surface of the HLA-B*35:08 molecule in white, with the LPEP peptide in cyan. The polymorphic residues are coloured in pink; A41, N80, R82, G83 and W167 in the HLA-B*35:08 molecule, correspond to T41, T80, L82, R83 and S167 in the HLA-B*44:02 molecule.

Figure S2. Analysis of SB27, CA5 and SB47 TCR binding to HLA-B35:08^{LPEP} and HLA-B*35:01^{LPEP}. The left and middle panels show the binding curves of immobilized SB27 (A), CA5 (B) and SB47 (C) TCRs with serial dilutions of HLA-B35:08^{LPEP} and HLA-B*35:01^{LPEP} in the fluid phase, respectively. The right panels show kinetic fits for the binding of SB27 (A), CA5 (B) and SB47 (C) TCRs with serial dilutions of HLA-B*35:08^{LPEP}.

Figure S3. Unbiased and refined density around the LPEP peptide. Unbiased (A and C) and refined (B and D) electron densities for the CA5 (A and B) and SB47 (C and D) ternary complexes. The unbiased electron density maps were generated via mFo-Fc maps at 3 sigma (green); refined density maps are contoured at 1 sigma with 2Fo-DFc maps (blue). The peptide is represented in black stick format.

Figure S4. Structural comparison of the SB47 TCR-pMHC-I complex. The N-terminally focused docking topology of the SB47 TCR (A) was structurally similar to the MHC-II-restricted autoimmune 3A6 TCR (B), the OB.1A12 TCR (C), and the E8 TCR (D). All MHC antigen binding clefts are coloured in white cartoon, with peptides shown as black sticks. CDR1α, purple; CDR2α, green; CDR3α, red; CDR1β yellow; CDR2β, sand; CDR3β, orange.

Table S1. Thermal stability assays of HLA B*35:08 mutants bound to the LPEP peptide.
Table S2. Surface plasmon resonance experiments for HLA-B*35:08^{LPEP} mutants and the SB27 and SB47 TCRs.
Table S3. Contact table for the CA5 TCR with the HLA-B*35:08^{LPEP} complex.
Table S4. Contact table for the SB47 TCR with the HLA-B*35:08^{LPEP} complex.

Table S1. Thermal stability assays of HLA B*35:08 mutants bound to the LPEP peptide.

HLA B*35:08 mutant-LPEP	Tm (°C)
B*35:08 wild-type-LPEP	60.3 ± 1.0
B*35:08-E55A-LPEP	57.3 ± 1.0
B*35:08-D61A-LPEP	59.0 ± 1.7
B*35:08-R62A-LPEP	59.8 ± 1.0
B*35:08-I66A-LPEP	48.5 ± 0.4
B*35:08-L163A-LPEP	57.0 ± 0.6
B*35:08-E166A-LPEP	57.6 ± 1.6
B*35:08-R170A-LPEP	57.1 ± 1.4

Tm, or thermal melt, is the temperature required to reach 50% unfolded protein.

Table S2. Surface plasmon resonance experiments for HLA-B*35:08^{LPEP} mutants and the SB27 and SB47 TCRs.

HLA-B*35:08 mutant-LPEP	SB27 TCR Kd_{eq} (μM)	SB47 TCR Kd_{eq} (μM)
B*35:08 wild-type-LPEP	10.4 ± 1.6	22.3 ± 1.6
B*35:08-E55A-LPEP	12.6 ± 0.6	NB
B*35:08-D61A-LPEP	11.7 ± 0.1	NB
B*35:08-R62A-LPEP	17.5 ± 7.3	NB
B*35:08-I66A-LPEP	60.8 ± 10.0	160.6 ± 24.7
B*35:08-R151A-LPEP	87.1 ± 6.2	28.9 ± 2.4
B*35:08-Q155A-LPEP	48.1 ± 5.6	25.7 ± 0.7
B*35:08-L163A-LPEP	16.1 ± 3.4	>200
B*35:08-E166A-LPEP	18.5 ± 0.5	162 ± 5.7
B*35:08-R170A-LPEP	5.4 ± 0.4	NB

Equilibrium dissociation constants (Kd_{eq}) were determined from duplicate measurements for SB27 and SB47 TCR binding to HLA-B*35:08 wild-type and mutants bound to the LPEP peptide. The Kd_{eq} values represent the mean ± standard error of the mean (sem).

Table S3. Contact table for the CA5 TCR with the HLA-B*35:08^{LPEP} complex.

TCR gene segment	CA5 TCR	HLA-B*35:08	Type of bond
CDR1α	Thr^{36}	Ala^{158}, Gly^{162}	VDW
CDR1α	Thr^{36}O-γ1	Ala^{158}O	H-bond
CDR2α	Asn^{57}	Glu^{154}	VDW
CDR2α	Asn^{57}N-δ2	Glu^{154}O-ε1	H-bond
CDR2α	Phe^{59}	Glu^{154}, Arg^{157}, Ala^{158}, Glu^{161}	VDW
CDR3α-N	Phe^{110}	Gln^{155}, Ala^{158},	VDW
CDR3α-N	Phe^{110}O	Gln^{155}N-ε2	H-bond
CDR3α-J	Tyr^{111}	Gln^{155}, Ala^{158}, Tyr^{159}, Leu^{163}	VDW
CDR3β-N	Glu^{109}	Ala^{150}, Arg^{151}	VDW
CDR3β-J	Thr^{110}	Arg^{151}	VDW
**TCR gene segment**	**CA5 TCR**	**LPEP peptide**	**Type of bond**
CDR1α	Tyr^{38}	Gln^{7}	VDW
CDR1α	Tyr^{38}OH	Gln^{7}Nε1	H-bond
CDR3α-V	Ser^{108}	Gln^{7}	VDW
CDR3α-V	Ser^{108}O-γ	Gln^{7}N-ε2	H-bond
CDR3α-N	Gly^{109}	Gln^{7}	VDW
CDR3α-N	Phe^{110}	Pro^{6}, Gln^{7}	VDW
CDR3α-J	Tyr^{111}	Pro^{4}, Leu^{5}, Gln^{7}	VDW
CDR3α-J	Asn^{112}	Leu^{5}, Pro^{6}, Gln^{7}	VDW
CDR3α-J	Asn^{112}N	Leu^{5}O	H-bond
CDR3α-J	Asp^{114}	Gln^{7}	VDW
CDR1β	Met^{27}	Gln^{9}	VDW
CDR1β	Asn^{28}	Gln^{9}	VDW
CDR1β	Asn^{28}N-δ2	Gln^{9}N-ε2	H-bond
CDR1β	His^{29}	Gln^{7}, Gly^{8}, Gln^{9}	VDW
CDR1β	Asn^{37}	Pro^{6}, Gln^{7}, Gly^{8}	VDW
CDR1β	Asn^{37}N	Gln^{7}O, Gly^{8}O	H-bond
CDR1β	Asn^{37}N-δ2	Pro^{6}O, Gly^{8}O	H-bond
CDR1β	Ser^{38}	Gln^{7}	VDW
CDR1β	Ser^{38}N	Gly^{7}O	H-bond
FWβ	Tyr^{40}	Gln^{7}	VDW
FWβ	Tyr^{40}OH	Gln^{7}N-ε2	H-bond
CDR3β-N	Pro^{107}	Gln^{7}, Gly^{8}	VDW

CDR: complementarity-determining region; FW: framework; V: variable; J: junction; N: non-germline; VDW: van der Waals; H-bond: hydrogen bond.

Table S4. Contact table for the SB47 TCR with the HLA-B*35:08^{LPEP} complex.

TCR gene segment	SB47 TCR	HLA-B*35:08	Type of bond
CDR1α	Asp^{37}	Trp^{167}, Arg^{170}	VDW
CDR1α	Asp^{37}O-δ1,δ2	Arg^{170} NH1, NH2	Salt bridge
CDR2α	Leu^{57}	Leu^{163}	VDW
CDR2α	Ser^{58}	Gly^{162}, Glu^{166}	VDW
CDR2α	Asn^{59}	Glu^{166}	VDW
CDR2α	Asn^{59}N	Glu^{166}O-ε1	H-bond
FWα	Thr^{82}	Glu^{166}	VDW
CDR3α-N	Gly^{109}	Glu^{55}, Gly^{56}, Arg^{170}	VDW
CDR3α-N	Gly^{109}O	Gly^{56}N	H-bond
CDR3α-J	Ser^{110}	Gly^{56}, Pro^{57}, Glu^{58}, Tyr^{59}, Trp^{167}, Arg^{170}	VDW
CDR3α-J	Ser^{110}O	Glu^{58}N	H-bond
CDR3α-J	Ser^{110}O-γ	Glu^{55}O-ε1	H-bond
CDR3α-J	Asn^{111}	Pro^{57}, Glu^{58}	VDW
CDR3α-J	Tyr^{112}	Pro^{57}, Glu^{58}, Asp^{61}	VDW
CDR3α-J	Tyr^{112}OH	Asp^{61}O-δ2	H-bond
CDR2β	Tyr^{57}	Asp^{61}, Gln^{65}	VDW
CDR2β	Glu^{58}O-ε1	Lys^{68}Nς	Salt bridge
FWβ	Arg^{66}	Pro^{57}, Asp^{61}	VDW
FWβ	Arg^{66}N-ε	Asp^{61}O-δ1, δ2	H-bond
FWβ	Arg^{66}NH2	Pro^{57}O	H-bond
FWβ	Arg^{66}NH1, NH2	Asp^{61}O-δ1, δ2	Salt bridge
FWβ	Gln^{67}	Pro^{57}	VDW
CDR3β-N	Thr^{109}	Gln^{65}, Asp^{61}	VDW
CDR3β-N	Gly^{110}	Arg^{62}, Ile^{66}	VDW
CDR3β-N	Gly^{110}O	Arg^{62} NH1	H-bond
CDR3β-J	Ser^{111}	Arg^{62}	VDW
CDR3β-J	Ser^{111}O	Arg^{62}NH1, NH2	H-bond
CDR3β-J	Tyr^{113}	Arg^{62}	VDW
CDR3β-J	Tyr^{113}OH	Arg^{62} NH1	H-bond
**TCR gene segment**	**SB47 TCR**	**LPEP peptide**	**Type of bond**
CDR3β-N	Arg^{108}	Leu^{5}, Pro^{6}, Gln^{7}	VDW
CDR3β-N	Arg^{108}NH2	Pro^{6}O, Gln^{7}O, Gly^{8}O	H-bond
CDR3β-N	Thr^{109}	Leu^{5}	VDW
CDR3β-N	Gly^{110}	Pro^{4}, Leu^{5}	VDW
CDR3β-N	Gly^{110}O	Leu^{5}N	H-bond
CDR3β-J	Ser^{111}	Pro^{4}, Leu^{5}, Pro^{6}, Gln^{7}	VDW
CDR3β-J	Ser^{111}O-γ	Leu^{5}O, Pro^{6}O	H-bond
CDR3β-J	Thr^{112}	Leu^{5}, Gln^{7}	VDW
CDR3β-J	Tyr^{113}	Leu^{1}	VDW
CDR3β-J	Glu^{114}	Gln^{7}	VDW

CDR: complementarity-determining region; FW: framework; V: variable; J: junction; N: non-germline; VDW: van der Waals; H-bond: hydrogen bond.

Supplementary Figure 1.

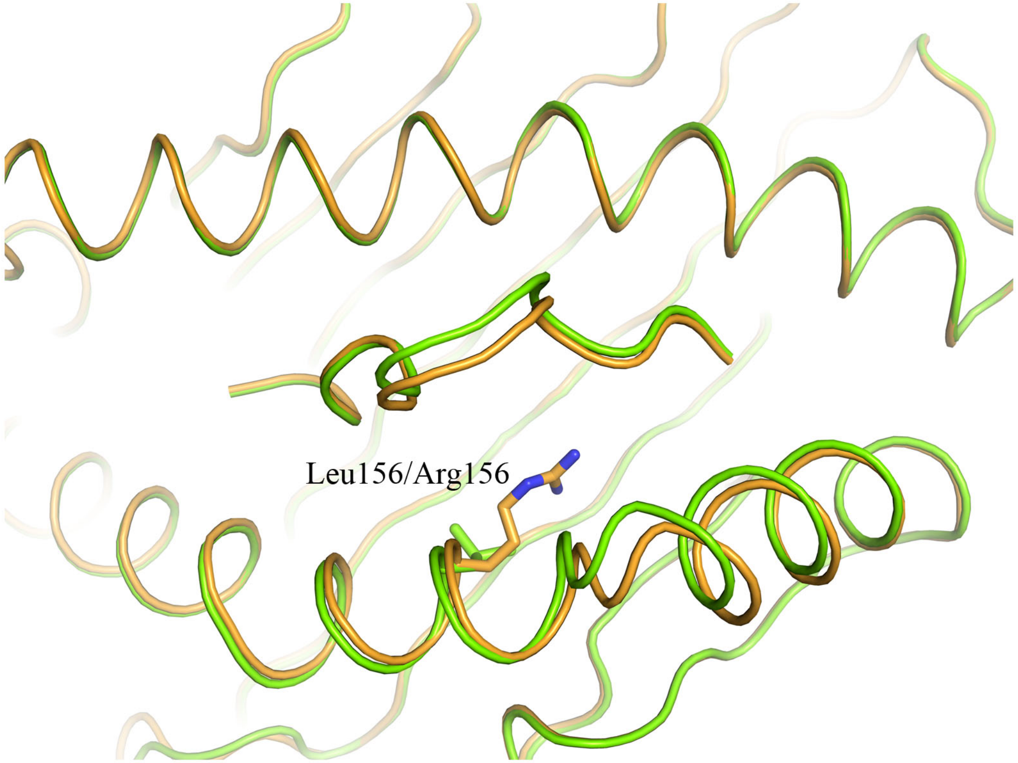

A

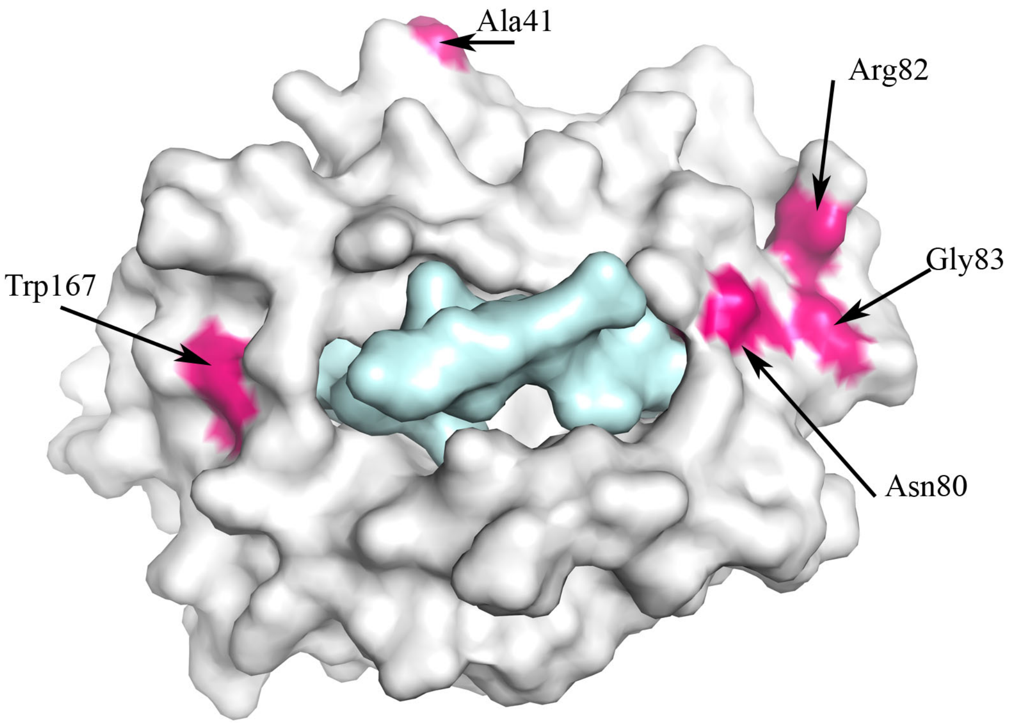

B

Supplementary Figure 2.

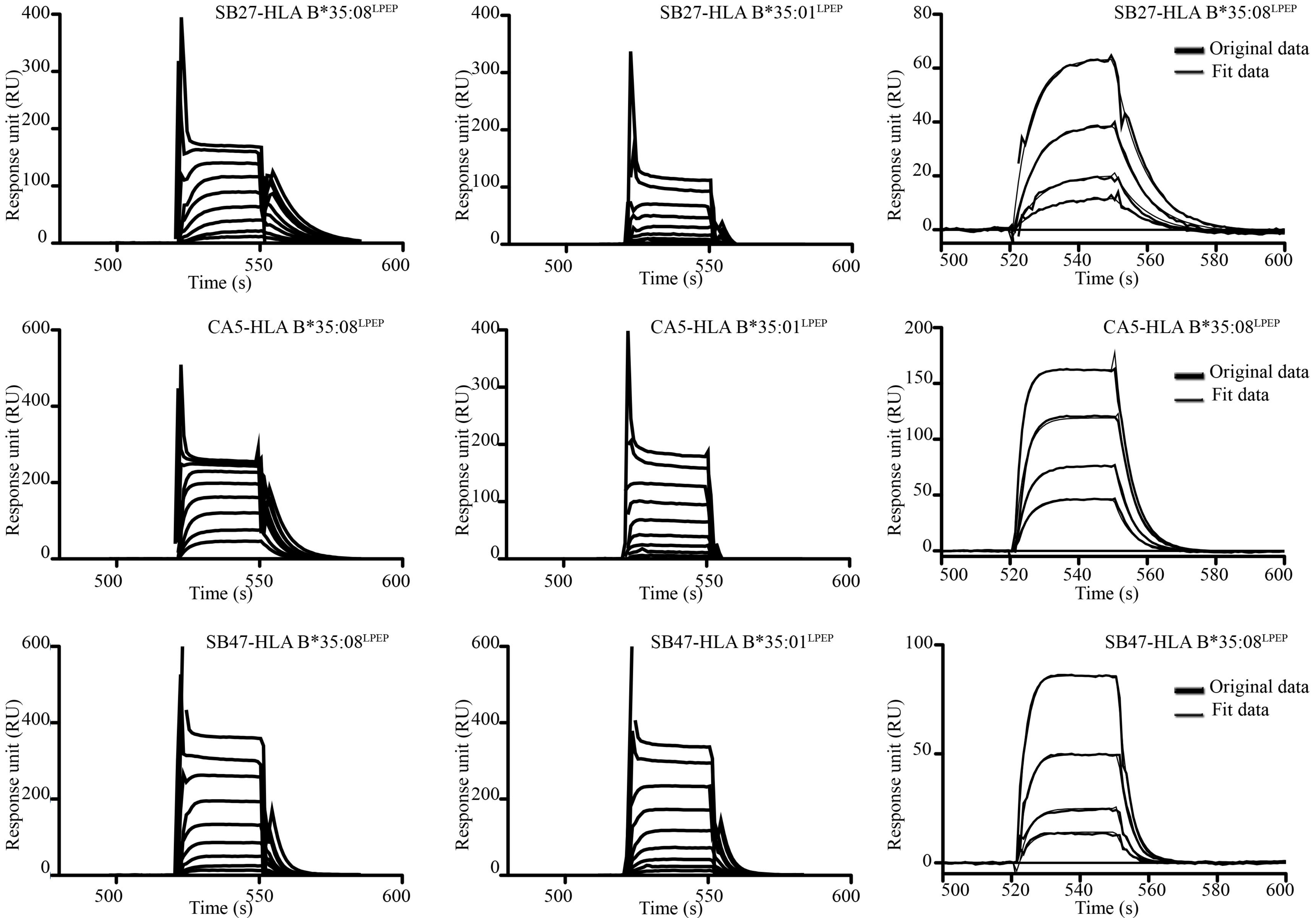

Supplementary Figure 3.

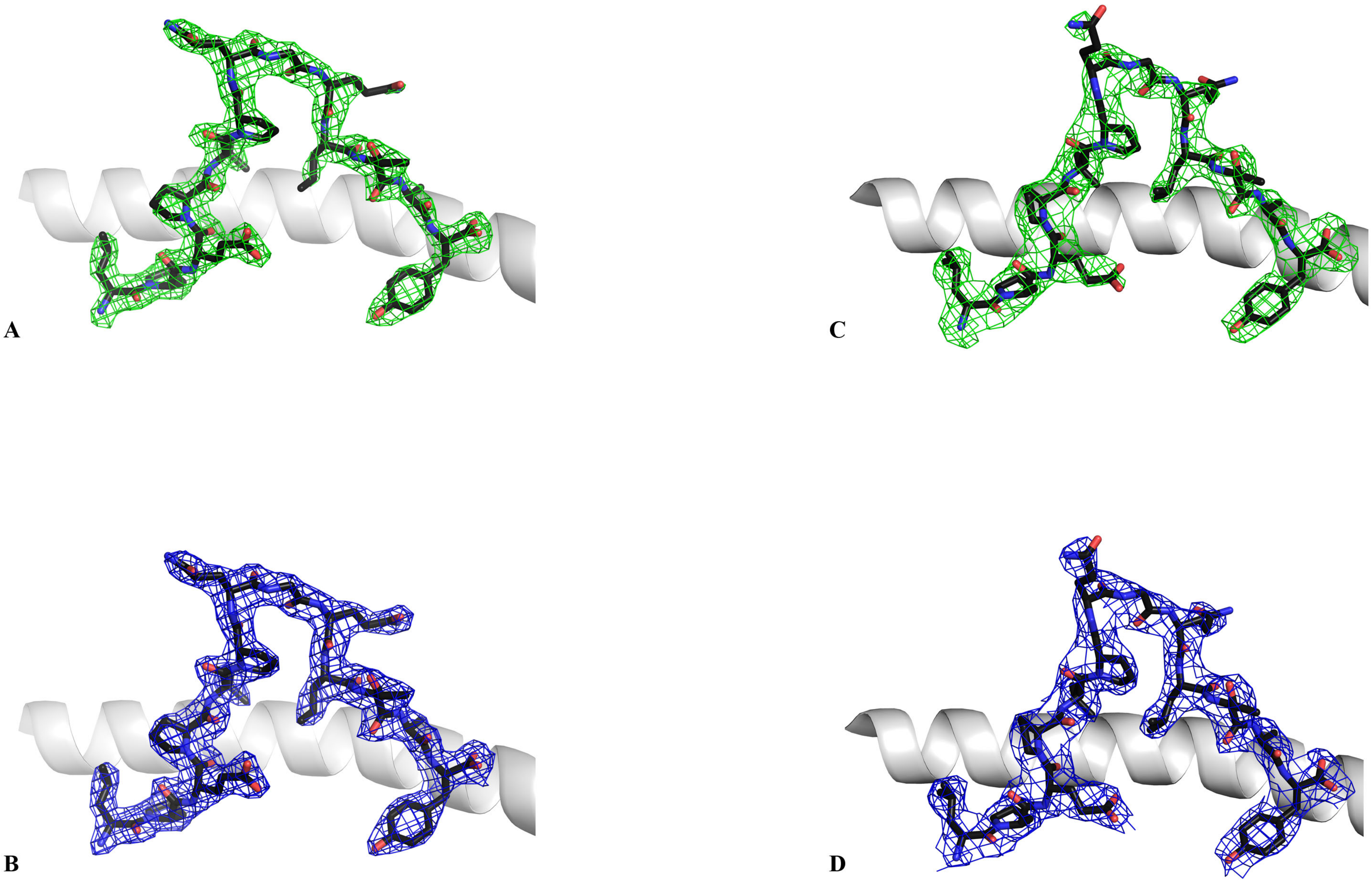

Supplementary Figure 4.

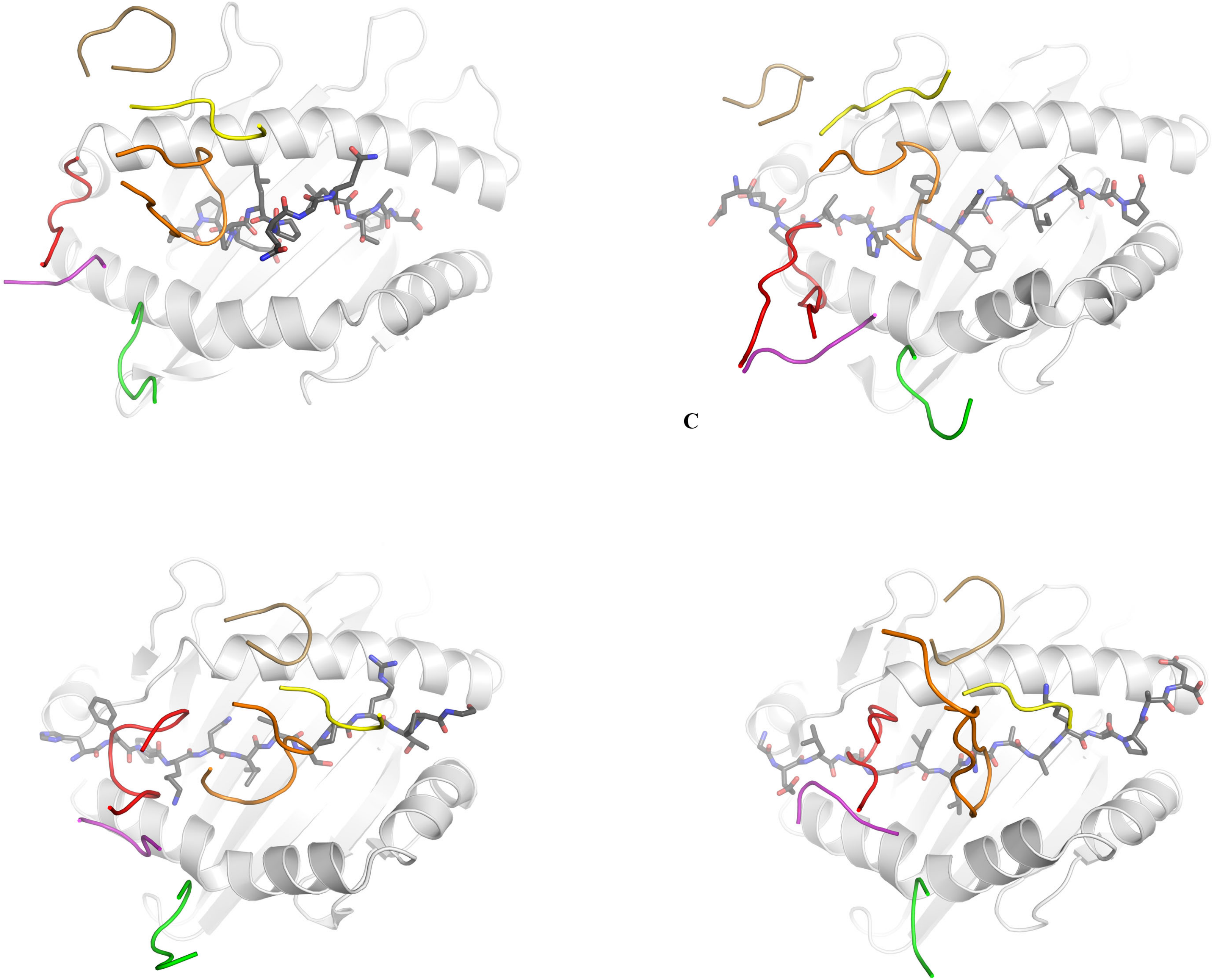

A

B

C

D
